# Targeting Arginine in COVID-19-Induced Immunopathology and Vasculopathy

**DOI:** 10.3390/metabo12030240

**Published:** 2022-03-11

**Authors:** William Durante

**Affiliations:** Department of Medical Pharmacology and Physiology, University of Missouri, Columbia, MO 65212, USA; durantew@health.missouri.edu

**Keywords:** COVID-19, arginine, arginase, nitric oxide synthase, immunopathology, endothelial dysfunction, thrombosis, vascular disease

## Abstract

Coronavirus disease 2019 (COVID-19) represents a major public health crisis that has caused the death of nearly six million people worldwide. Emerging data have identified a deficiency of circulating arginine in patients with COVID-19. Arginine is a semi-essential amino acid that serves as key regulator of immune and vascular cell function. Arginine is metabolized by nitric oxide (NO) synthase to NO which plays a pivotal role in host defense and vascular health, whereas the catabolism of arginine by arginase to ornithine contributes to immune suppression and vascular disease. Notably, arginase activity is upregulated in COVID-19 patients in a disease-dependent fashion, favoring the production of ornithine and its metabolites from arginine over the synthesis of NO. This rewiring of arginine metabolism in COVID-19 promotes immune and endothelial cell dysfunction, vascular smooth muscle cell proliferation and migration, inflammation, vasoconstriction, thrombosis, and arterial thickening, fibrosis, and stiffening, which can lead to vascular occlusion, muti-organ failure, and death. Strategies that restore the plasma concentration of arginine, inhibit arginase activity, and/or enhance the bioavailability and potency of NO represent promising therapeutic approaches that may preserve immune function and prevent the development of severe vascular disease in patients with COVID-19.

## 1. Introduction

Severe acute respiratory syndrome coronavirus 2 (SARS-CoV-2) is the causative agent of the coronavirus disease 2019 (COVID-19) pandemic. This disease is a substantial threat to human health with over 418 million cases worldwide and over 5.8 million confirmed deaths, as of February 2022 [1]. SARS-CoV-2 is transmitted primarily by respiratory droplets; however, direct aerosol contact with contaminated sources and fecal–oral transmission are also possible [2]. The virus infects the host by targeting airway and alveolar epithelial cells in the lung that express the surface receptor angiotensin-converting enzyme 2 (ACE2). The coronavirus enters host cells via its surface spike protein that binds to ACE2 through its receptor binding domain where it is proteolytically activated by human proteases allowing for cell entry. Subsequent viral replication and release causes the host cell to undergo pyroptosis and emit damage-associated molecular patterns, including ATP and nucleic acid, which triggers the discharge of proinflammatory cytokines and chemokines. However, not all exposures to SARS-CoV-2 lead to symptomatic infection. Among infected individuals that develop symptoms, an estimated 80–85% experience mild flu-like symptoms, such as fever, cough, myalgia, and fatigue, and most recover in a few days or weeks [3]. The remaining 15–20% of patients suffer more severe symptoms that may require hospitalization and treatment in an intensive care unit. Overall mortality rates vary greatly depending on risk factors but lie between 0.3 and 3.0% of all infected individuals [4]. The markedly heterogenous presentation of COVID-19 likely reflects the degree of viral infection and the activity of the host’s immune system [5]. In most cases, the destruction of lung cells by SARS-CoV-2 initiates a local immune response that promotes the release of anti-viral cytokines and primes adaptive T and B cell immune responses, leading to the resolution of the infection. Alternatively, in some instances, a dysfunctional immune response occurs where a proinflammatory feedback loop is established, eliciting a cytokine storm that mediates widespread lung inflammation resulting in severe pneumonia and acute respiratory distress syndrome (ARDS). Moreover, this heightened systemic inflammatory state induces endothelial cell (EC) dysfunction and vascular smooth muscle cell (SMC) proliferation and migration, precipitating a constellation of vascular complications, such as stroke, ischemia, and thrombosis, which contribute to multi-organ failure and the high mortality rate in COVID-19 [6].

The metabolism of arginine serves as a key regulator of innate and adaptive immunity [7,8,9,10,11]. Arginine catabolism in myeloid cells is largely driven by nitric oxide (NO) synthase (NOS) and arginase (ARG), and the differential regulation of these enzymes augments or diminishes the immune response. In a similar fashion, the divergent modulation of the activity of these two enzymes dictates vascular cell function, where NOS serves to maintain vascular health while ARG is linked to EC dysfunction and vascular disease [12,13,14,15,16,17,18]. Intriguingly, ARG expression is upregulated in patients with COVID-19 in a disease-dependent manner, suggesting that the rewiring of arginine metabolism by ARG may contribute to poor outcomes in COVID-19 patients [19]. In this review, we highlight the alteration in arginine metabolism during SARS-CoV-2 infection and discuss how the induction of ARG may contribute to immune and vascular cell dysfunction and its attendant risk of life-threatening respiratory and vascular complications. In addition, it explores potential therapeutic applications that target arginine in COVID-19.

## 2. Overview of Arginine Metabolism

**Figure 1:**
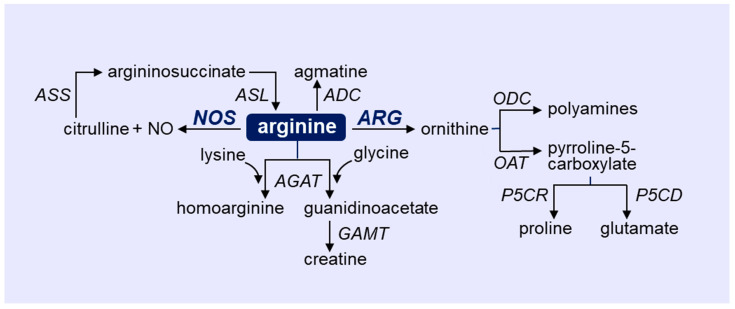
Outline of arginine metabolism via four distinct enzymatic pathways. Arginine catabolism in immune and vascular cells is largely driven by the enzymes nitric oxide synthase (NOS) and arginase (ARG). ADC, arginine decarboxylate; AGAT, arginine-glycine amidinotransferase; ASL, argininosuccinate lyase; ASS, argininosuccinate synthetase; GAMT, guanidinoacetate N-methyltransferase; ODC, ornithine decarboxylase; OAT ornithine aminotransferase; P5CR, pyrroline-5-carboxylate reductase; P5CD, pyrroline-5-carboxylate dehydrogenase.

Arginine is a cationic, semi-essential amino acid that plays an important role in regulating immune and vascular cell function [12,13]. Levels of free arginine within the body are derived from the diet, endogenous synthesis, and turnover of proteins. In healthy adults, endogenous synthesis is sufficient so that arginine is not an essential amino acid. However, in cases of infection where catabolic stress occurs, arginine becomes conditionally essential as endogenous synthesis is inadequate to meet increases in metabolic demand. Arginine is involved in the synthesis of proteins and the removal of ammonia by the urea cycle in the liver, and serves as a precursor for several molecules, including NO, citrulline, proline, glutamate, polyamines, creatinine, agmatine, and homoarginine (Figure 1). Arginine is metabolized to NO and citrulline by NOS [12,13,14,15]. Aside from functioning as a substrate for the enzyme, arginine aids in the intracellular assembly of the functional dimeric form of NOS and contributes to the proper coupling between the reductive and oxidative domains of the enzyme. Accordingly, the absence of arginine results in the uncoupling of the enzyme and the generation of superoxide rather than NO. The NOS product citrulline is subsequently recycled back to arginine by the serial action of argininosuccinate synthetase (ASS) and lyase. ASS is the rate-limiting enzyme in this salvage pathway, and it tightly controls NOS-derived NO synthesis [20]. There are three distinct isoforms of NOS: neuronal NOS (nNOS or NOS1), inducible NOS (iNOS or NOS2), and endothelial NOS (eNOS or NOS3). nNOS and eNOS are constitutively expressed as calcium-dependent enzymes that transiently release NO in responsive to specific physiologic stimuli. In contrast, iNOS is a calcium-insensitive protein that is induced by proinflammatory cytokines and microbial-associated products. Once formed, iNOS generates large amounts of NO over a prolonged period. While nNOS-derived NO is implicated in synaptic plasticity and serves as a neurotransmitter for both the central and peripheral nervous system, NO generated by the high-output iNOS enzyme plays a critical role in host defense, exerting cytotoxic effects on bacteria, parasites, viruses, and tumor cells [21]. In addition, iNOS-derived NO contributes to the pathophysiology of inflammatory disease and is the predominant mediator of hypotension in septic shock. Alternatively, eNOS functions to maintain vascular health. The basal release of NO by ECs promotes blood flow by inhibiting arterial tone. In addition, the luminal release of NO elicits a potent antithrombotic effect by inhibiting blood coagulation and platelet activation, adhesion, and aggregation, while the abluminal liberation of the gas limits the intimal thickening of blood vessels by blocking vascular SMC proliferation, migration, and extracellular matrix deposition. EC-derived NO also prevents inflammation by retarding the synthesis of inflammatory cytokines and chemokines; the expression of surface adhesion receptors; and the recruitment, infiltration, and activation of leukocytes within the vasculature. In contrast, the loss of NO production causes endothelial dysfunction that is symbolized by impaired endothelium-dependent vasodilation, EC activation and apoptosis, endothelial barrier disruption, arterial stiffness, vessel wall thickening, and a prothrombotic and inflammatory state.

Arginine is also hydrolyzed to urea and ornithine by the manganese metalloenzyme ARG. There are two isoforms of ARG, ARG1 and ARG2, which are encoded by different genes mapped on separate chromosomes, but they share approximately 60% amino acid sequence homology. Although they possess a similar mechanism of arginine metabolism, these isozymes differ in their tissue distribution, subcellular localization, and molecular regulation [22]. ARG1 is a cytosolic enzyme that is highly expressed in the liver where it catalyzes the final step of the urea cycle. ARG1 is also found outside the liver in various tissues, including myeloid cells. Alternatively, ARG2 is a mitochondrial enzyme that is commonly expressed in extrahepatic tissues, most prominently in the kidney. While ARG1 plays a fundamental role in inflammation-associated immunosuppression, both ARG isoforms have been linked to vascular disease by triggering EC dysfunction [10,11,12,13,14,15]. ARG-derived urea is readily excreted by the kidneys while ornithine is further metabolized by ornithine decarboxylase (ODC) to putrescine and the downstream polyamines, spermine, and spermidine [23]. Ornithine is also catabolized by ornithine aminotransferase (OAT) to pyrroline-5-carboxylate, which is, in turn, converted to proline by pyrroline-5-carboxylate reductase or to glutamate by pyrroline-5-carboxylate dehydrogenase. While polyamines play an essential role in cell growth, proline is used for the synthesis of many structural proteins, especially collagen, which is involved in fibrosis [12,13,24,25,26]. Arginine is also metabolized by arginine:glycine amidinotransferase to produce homoarginine or guanidinoacetate, and the latter is converted to creatine by N-methyltransferase. Finally, arginine may be catabolized by arginine decarboxylase to agmatine, which is converted to putrescine by agmatinase. However, the presence and functional significance of arginine decarboxylase in immune and vascular cells remains to be established.

There is substantial crosstalk between the two main arginine metabolizing enzymes: NOS and ARG. By restricting the availability of arginine, ARG promotes the uncoupling of NOS, thereby diminishing NO synthesis and elevating superoxide generation [27]. In addition, arginine depletion by ARG limits the translation of iNOS by activating the general control nonderepressible 2 (GCN2) kinase, while ARG-derived spermine inhibits the expression of iNOS, leading to further reductions of NO production [28,29]. Finally, the NOS-derived intermediate product N-ω-hydroxy-L-arginine directly inhibits ARG activity, whereas iNOS-formed NO selectively stimulates ARG1 activity by nitrosylating cysteine residues of the protein [30,31]. Thus, these two arginine-metabolizing enzymes show reciprocal and regulatory interactions that impact their activity.

## 3. Role of NOS and ARG in Immune Cells

Considerable evidence has established an important role for iNOS and ARG1 in the modulation of immune responses via the catabolism of arginine. Macrophages display distinct phenotypic heterogeneity and canonical classifications divide activated macrophages into two functional subsets: M1 or classically activated and M2 or alternatively activated [11]. However, this grouping is an oversimplification as macrophages exist in a continuum between these two functional states. M1 macrophages largely consume arginine via iNOS. In these macrophages, iNOS is induced by the T helper 1 (Th1) cytokines interferon (IFN), interleukin-1 (IL-1), and tumor necrosis factor-α (TNFα), which are mobilized in the initial phase of the immune response to pathogen infection. The Th1 cytokines also simultaneously stimulate the expression of arginine transporters and enzymes associated with the synthesis of iNOS co-factors to maximize and sustain NO synthesis. The generation of NO bestows M1 macrophages with potent proinflammatory and microbiocidal properties, allowing for the cytotoxic clearing of viruses, bacteria, fungi, protozoa, and tumor cells. NO has antiviral effects against several viruses, including herpes simplex virus, Epstein–Barr virus, the poxviruses ectromelia and vaccinia, and herpes simplex virus [32,33,34]. In addition, endogenous production of NO by iNOS blocks the replication of the RNA enterovirus coxsackie B3 [35]. The expression of iNOS is significantly elevated in the heart and spleen of mice infected with coxsackie B3 virus; however, the pharmacological inhibition of iNOS significantly increases viral load and mortality in these animals. In this case, NO nitrosylates and inactivates the viral protease 3C, which is necessary for replication of the virus [36]. Significantly, NO has been reported to interfere with the replication cycle of SARS-CoV-1 via two distinct mechanisms [37,38]. First, NO reduces the palmitoylation of nascently expressed spike protein which disrupts the interaction between the spike protein and its cognitive receptor, ACE2. Second, NO limits RNA production in the early steps of viral replication through chemical modification/inactivation of the cysteine proteases encoded by Orf1a of SARS-CoV-1. Given the high degree of homology between SARS-CoV-1 and SARS-CoV-2 proteins, NO likely mitigates SARS-CoV-2 replication in a similar fashion [39,40].

M2 macrophages primarily metabolize arginine via ARG1. In these cells, T helper 2 (Th2) cytokines, such as IL-4 and IL-13, stimulate the expression of ARG1, which serves as an important hallmark of M2 differentiation. While ARG2 is also detected in macrophages, its biological role in these cells is not known [41]. M2 macrophages are involved in the second phase of the immune response to pathogen invasion and serve to dampen inflammation by redirecting arginine away from iNOS and stimulate tissue repair via the ARG1-mediated generation of polyamines and proline [42]. ARG and iNOS are also expressed in dendritic cells and they have been linked to the function of subsets of dendritic cells that arise in response to local environmental cues. A population of TNFα and iNOS-producing dendritic cells (Tip-DCs) have been identified that exert proinflammatory actions and promote resistance to several, but not all pathogens [7]. Conversely, silencing ARG2 expression in dendritic cells is a prerequisite for their maturation and ability to induce optimal T cell priming [43].

ARG is expressed in polymorphonuclear neutrophils and myeloid-derived suppressor cells (MDSCs) [7,10]. Notably, these cells secrete ARG1 in the extracellular milieu, leading to local arginine depletion, which is critically involved in the suppression of T cell function [44,45,46]. Indeed, culturing T cells in medium with reduced arginine levels markedly impairs T cell function, whereas the incubation of T cells in a high arginine environment enhances their function [47,48]. Moreover, the de novo synthesis of arginine following the addition of citrulline rescues T cell function in an ASS-dependent manner [49]. Although early studies suggested that T cell dysfunction was due to a reduction in the CD3ζ subunit of the T cell receptor (TCR) complex, arginine-starved cells produce IL-2 and upregulate the early activation markers CD25, CD 69, CD122, and CD132, indicating that the effect induced by arginine deprivation is not caused by a defect in TCR signaling [50]. Instead, T cells cultured in arginine-free media are arrested in the G_0_/G_1_ phase of the cell cycle due to impaired expression of cyclin D3 and cyclin-dependent kinase 4 (cdk4) through decreases in mRNA stability and protein translation [51,52]. In fact, T cells grown in arginine-depleted media suffer from a global decrease in translation secondary to the GCN2 kinase-mediated phosphorylation/inactivation of the translation initiation factor eIF2α. Recent work also suggests a potential role of rictor–mammalian target of rapamycin complex 2 (mTORC2) in regulating the suppression of T cell responses by amino acid deprivation [52]. Furthermore, MDSCs may negatively impact the immune system via the generation of NO by NOS [53]. Aside from stimulating apoptosis, NO dampens T cell proliferation and differentiation by blocking IL-2 production and signaling [54,55]. Interestingly, the expression of both iNOS and ARG2 in T cells has been shown to impair their function [56,57]. This is consistent with a report demonstrating that intracellular arginine levels directly promote the metabolic fitness and survival of T cells [48]. Arginine availability and metabolism also modifies B lymphocyte biology as decreases in plasma arginine following ARG1 overexpression impair the developmental transition from pro- to pre-B cells in bone marrow, leading in lowered B cell cellularity in secondary lymphoid organs independent of any change in B cell proliferation and cytokine secretion [58]. In contrast, iNOS is an intrinsic factor for activated B cells and its activity is crucial for the survival of plasma cells [59]. Early work also found that arginine supplementation potentiates the cytotoxicity of both human and murine natural killer (NK) cells, whereas arginine starvation diminishes their toxicity [60,61,62,63]. In addition, the production of NO by eNOS protects NK from activation-induced cell death by regulating the expression of TNFα, while the cytokine-mediated expression of iNOS is involved in their cytotoxic actions [64,65]. Conversely, ARG1 activity secreted from human granulocytes and MDSCs suppress the function of NK cells [66,67]. 

## 4. Role of NOS and ARG in Vascular Cells

The endothelium, located in innermost layer of blood vessels, plays a fundamental role in preserving vascular health via the generation of NO by eNOS. Endothelial dysfunction and its associated reduction in NO bioavailability represents a seminal mechanism for the development of vascular disease and cardiovascular events [68,69]. The etiology of endothelial dysfunction is complex and multifactorial; however, emerging evidence indicates that ARG is a major mediator of EC malfunction. Both ARG isozymes are expressed in human ECs, and they effectively compete with eNOS for substrate arginine, leading to reductions in NO synthesis and elevations in superoxide formation secondary to the uncoupling of eNOS [12,13,14,15,16,17,18,70]. ARG expression is upregulated in ECs by several inimical stimuli, such as TNFα, lipopolysaccharide, oxidized low-density lipoprotein, high concentrations of glucose, uric acid, peroxynitrite, hypoxia, angiotensin II, and reactive oxygen species. Multiple signaling pathways for the induction of ARG have been identified; however, the p38 mitogen-activated protein kinase and small GTPase Rho both play a central role [15,18,71]. ARG activity is also increased by thrombin through activating protein-1, which then contributes to EC dysfunction in arterial thrombosis [72,73]. In addition, epigenetic mechanisms through histone deacetylation and DNA methylation, as well as posttranscriptional regulation by miRNA, have been reported in vascular cells [15,16,17,18]. Significantly, the induction of ARG has been implicated in the development of endothelial dysfunction in various cardiovascular pathologies, including systemic and pulmonary arterial hypertension, sickle cell disease, diabetes, atherosclerosis, trauma, obesity, aging, myocardial ischemia–reperfusion injury, and hemorrhagic shock [27,74,75,76,77,78,79,80,81,82,83]. Notably, ARG-mediated impairments of NO bioavailability and EC dysfunction are corrected by the pharmacological inhibition or genetic deletion of ARG in numerous experimental models, thus establishing this enzyme as a promising therapeutic target in treating vascular disease.

ARG is also a critical regulator of vascular SMC function. Overexpression of ARG1 stimulates SMC proliferation by increasing the production of polyamines, whereas pharmacological inhibition of ARG1 suppresses polyamine synthesis and SMC replication [84]. Consistent with these findings, our laboratory found that ARG1 promotes the entry of vascular SMCs into the cell cycle and that silencing ARG1 expression arrests SMCs in the G_0_/G_1_ phase of the cell cycle [85]. In addition, we observed that ARG1 stimulates collagen synthesis in SMCs by channeling the metabolism of arginine to proline [25,26]. We also discovered that ARG1 is upregulated following arterial injury and that it contributes to neointimal thickening [85]. Moreover, ARG1 causes arterial fibrosis and stiffening in hypertensive animals [86,87]. Elevated vascular ARG activity has also been implicated in the adverse vascular remodeling response observed in pulmonary arterial hypertension, aging, atherosclerosis, and obesity [27,74,88,89,90]. Thus, ARG plays a crucial role in promoting arterial lesion formation following injury and disease. 

## 5. Vascular Complications in COVID-19

Recent clinical data indicate that COVID-19 is associated with a significant risk of ischemia-related vascular disease. The rate of venous thromboembolism is markedly increased in patients with COVID-19 with pulmonary embolism being the most common thrombotic complication [91,92]. Moreover, a prospective cohort study found that pulmonary embolism was the direct cause of death in over 30% of patients, illustrating the critical interaction between COVID-19 and venous thrombosis [93]. Indeed, coagulation abnormalities, including elevated levels of circulating fibrin degradation products and von Willebrand factor, the prolongation of prothrombin time, and thrombocytopenia, are detected in hospitalized and severely ill patients with COVID-19 and may have prognostic value [94,95,96,97]. In addition, the rate of arterial thrombosis is elevated in COVID-19, which likely contributes to the greater incidence of myocardial infarction, ischemic stroke, and acute limb ischemia in this patient population [92,98,99,100]. There is also a strong association between COVID-19 with microvascular thrombosis. Autopsy findings reveal that platelet–fibrin thrombi are a common microscopic finding in the lungs of COVID-19 patients. Furthermore, the microvasculature of the lung is abnormal and characterized by acute endothelial injury, inflammation, and leaky and distorted capillaries [101,102]. The high incidence of thrombosis and vascular injury in the lungs may underlie the ventilation–perfusion mismatch and impaired oxygen uptake which exemplify the respiratory complications of COVID-19 [103]. Significantly, microthrombi are widely disseminated and found in the heart, kidney, and liver in patients with COVID-19, supporting the presence of multi-organ thrombotic microangiopathy in these patients [104].

Several mechanisms have been proposed to cause thrombosis in COVID-19 patients, including platelet activation and turnover; leukocyte activation; and the formation of neutrophil extracellular traps, complement system activation, coagulation defects, and endothelial dysfunction [6,105,106]. However, endothelial injury and malfunction evolve as a central pathological feature in COVID-19 [106,107,108,109]. Clinical signs of endothelial inflammation are widespread and seen in multiple organs, such as the lungs, heart, liver, kidney, intestine, and skin [101,110,111,112,113]. Post-mortem studies on lung samples from patients with COVID-19 also uncovered substantial EC damage, with evidence of apoptosis and loss of junctional integrity [101]. Furthermore, biomarkers of endothelial dysfunction are detected in the blood of patients with COVID-19 and appear to have prognostic relevance, being associated with severe disease [114,115,116]. The von Willebrand factor, a molecular marker of endothelial dysfunction, is increased in association with the development of ARDS following SARS-CoV-2 infection [114]. At the same time, circulating ECs, a cell-based marker of endothelial damage cast from injured blood vessels, is elevated in critically ill COVID-19 patients [117]. Circulating levels of P-selectin, E-selectin, soluble intercellular molecule-1, and angiopoietin-2, i.e., molecular markers of EC activation, are higher in patients with severe or fatal COVID-19 [118,119,120]. Although initial reports suggested that endothelial injury was caused directly by the virus, recent work favors an indirect mechanism mediated locally by an enhanced inflammatory response by infected airway epithelium and systemically by the excessive immune response to infection [107].

Accumulating data indicate that endothelium-dependent vasodilation is also compromised in COVID-19 patients. An initial case report found that endothelium-dependent microvascular reactivity of the skin is severely impaired in a patient with valvular heart disease and COVID-19 [121]. A subsequent follow-up study revealed that systemic endothelium-dependent vasodilation is reduced in both severe and mild-to-moderate COVID-19 patients compared to sex- and age-matched healthy volunteers [122]. Moreover, the decline in endothelial function is more pronounced in patients with severe COVID-19 and occurs in parallel with a rise in circulating proinflammatory cytokines and chemokines. More recently, flow-mediated dilation of the brachial artery was shown to be lower in young adults with SARS-CoV-2 and this was associated with higher arterial stiffness relative to healthy controls [123]. Collectively, these findings suggest that eNOS activity and NO bioavailability is diminished among COVID-19 patients. Interestingly, the endothelial glycocalyx senses biomechanical stimuli, triggering a host of intracellular events that lead to eNOS activation and NO release [124]; however, the endothelial glycocalyx is degraded in patients with COVID-19, providing a structural mechanism that limits NO production in response to flow [125,126]. Furthermore, the loss of the endothelial glycocalyx in COVID-19 patients may facilitate platelet adhesion, the infiltration of inflammatory cells into the vessel wall, and the release of endothelial products such as von Willebrand factor into the circulation. Finally, the inhibition of eNOS following SARS-CoV-2 infections is likely to be multifactorial as chronic inflammatory and oxidative stress have been implicated in this process [127].

## 6. Role of NOS and ARG in COVID-19

Emerging evidence indicates that arginine metabolism is altered in COVID-19 patients [128,129,130]. Early work revealed that circulating levels of NO are significantly higher in patients with severe COVID-19 [131]. This likely reflects the induction of iNOS in immune cells following virus infection, leading to local and systemic increases in NO that serve to combat the infection. However, as noted in the previous section, endothelial release of NO via eNOS is attenuated in COVID-19 patients, resulting in vasoconstriction and arterial and venous thrombosis. In addition, ARG1 is upregulated in whole blood, plasma, and peripheral blood mononuclear cells of patients with COVID-19 and may be a valuable diagnostic marker of the disease [132,133,134]. The expansion of MDSCs seen in COVID-19 directly correlates to elevated ARG activity and lymphopenia [135]. Monocytic MDSC growth is strikingly associated with COVID-19 disease severity and purified MDSCs block T cell proliferation, in part, via an ARG1-dependent mechanism, supporting a role for these cells in the aberrant COVID-19 immune response [19]. Moreover, granulocytic MDSCs express and secrete high levels of ARG1 protein that effectively depletes arginine from the local environment [136]. In fact, the expansion of MDSCs contributes to platelet activation by arginine deprivation during SARS-CoV-2 infection [137]. In addition, the expression of the prothrombotic GPIIb/IIIa complex is elevated on platelets from severe COVID-19 patients compared to healthy controls and inversely correlates with plasma arginine concentration. Together, these findings suggest that overexpression of ARG1 contributes to COVID-19-mediated immunopathology and vasculopathy.

Multiple metabolomic studies have documented a decrease in plasma arginine in patients with COVID-19 [138,139,140]. Similarly, a recently completed prospective observational study found that mean plasma arginine levels are lower among adult and pediatric patients with COVID-19 compared to healthy controls [141]. In addition, the arginine to ornithine ratio is low for adult and pediatric patients, indicating an elevation of ARG activity in these patients. Both patient groups also had a low global arginine bioavailability ratio, a known risk factor for major adverse cardiovascular events [142]. Another clinical study confirmed the decrease in circulating arginine and global arginine bioavailability ratio in severely ill COVID-19 patients [143]. Notably, these patients also had a two-fold increase in circulating levels of asymmetric dimethylarginine, a specific NOS inhibitor. Thus, COVID-19 may block eNOS-derived NO formation by limiting both substrate availability and enzyme activity. 

## 7. Targeting Arginine in COVID-19

**Figure 2:**
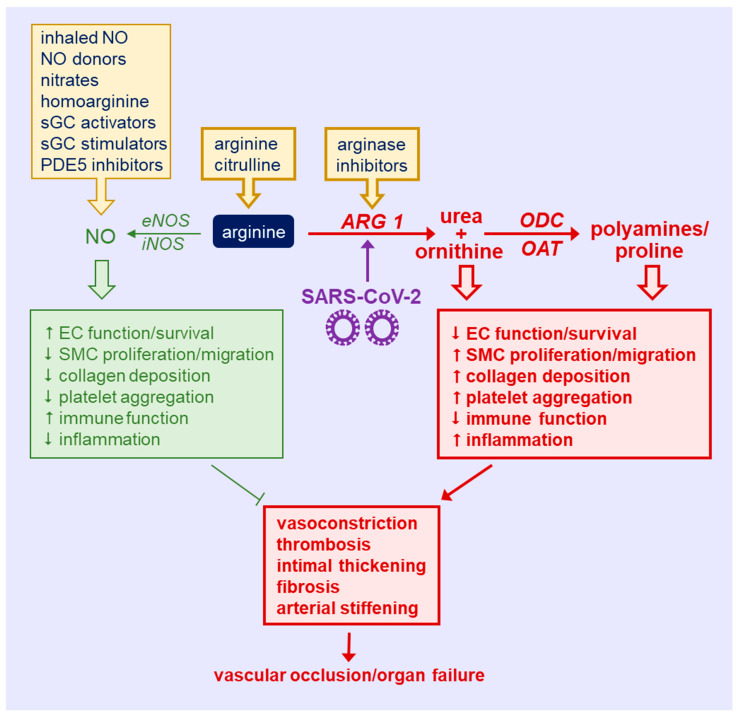
Targeting arginine in COVID-19-induced immune and vascular dysfunction. There is an upregulation of arginase 1 (ARG1) by the severe acute respiratory syndrome coronavirus 2 (SARS-CoV-2) that diminishes circulating arginine levels and shunts the metabolism of arginine away from the synthesis of nitric oxide (NO) by NO synthase toward the production of ornithine, which is subsequently converted to polyamines and proline via the action of ornithine decarboxylase (ODC) and ornithine aminotransferase (OAT), respectively. The induction of ARG1 causes immune malfunction, inflammation, and endothelial cell (EC) death and dysfunction, and stimulates vascular smooth muscle cell (SMC) proliferation, migration, collagen synthesis, and platelet aggregation, leading to vasoconstriction, thrombosis, arterial thickening, fibrosis, and stiffening. Collectively, these actions will promote vascular occlusion and organ failure. Several strategies may be used to target arginine in COVID-19. Dietary supplementation with arginine or citrulline provides a forthright approach to restore circulating levels of arginine in SARS-CoV-2-infected patients. Alternatively, the use of ARG inhibitors provides a more selective modality in correcting disturbances of arginine metabolism in COVID-19. In addition, the direct administration of arginine metabolites (inhaled NO, NO donors, inorganic nitrates, and homoarginine) or NO-potentiating drugs [soluble guanylate cyclase (sGC) activators or stimulators, and phosphodiesterase type 5 (PDE5) inhibitors] affords another avenue in treating COVID-19 patients. *eNOS*, endothelial nitric oxide synthase; *iNOS*, inducible nitric oxide synthase.

There is a growing appreciation for the role of amino acids in regulating immune and vascular cell function. Substantial evidence suggests that the metabolism of arginine is altered in COVD-19. In particular, the bioavailability of arginine is seriously compromised in COVID-19 patients and there is an upregulation of ARG1 that skews the metabolism of arginine away from the synthesis of NO (Figure 2). By limiting the production of NO, ARG1 depresses EC survival and function, immune responses, and augments platelet aggregation and inflammation, leading to vasoconstriction and thrombosis. In addition, the reduction in NO synthesis, coupled with the ARG1-mediated shunting of arginine toward polyamine and proline synthesis, will stimulate vascular SMC proliferation, migration, and collagen deposition (resulting in arterial thickening), fibrosis, and stiffening. Collectively, these actions will promote vascular occlusion and organ failure.

Several strategies can be employed to correct the disturbances of arginine metabolism in COVID-19. One straightforward approach involves amino acid therapy to target the deficiency of arginine in patients with COVID-19. In this regard, numerous experimental and clinical studies have shown that enteral or parenteral administration of arginine ameliorates endothelial function in a host of vascular diseases [12,144,145]. Of concern, arginine is a key nutrient in the lifecycle of many viruses, and the replenishment of this amino acid may stimulate SARS-CoV-2 replication. Indeed, arginine depletion has been proposed as a potential treatment for COVID-19 [146,147]. However, given that circulating levels of arginine are already low in COVID-19, a further reduction may potentially exacerbate the illness. Moreover, interim results from a randomized clinical trial found that the addition of arginine to standard therapy in patients hospitalized with COVID-19 reduced respiratory support and in-hospital stay relative to placebo-treated controls, supporting the use of arginine in the treatment of COVID-19 [148]. Of note, the dose of arginine (1.66 g twice per day) used in the study was rather low and it is not known whether it increased arginine availability in these patients. Owing to the extensive metabolism of orally administered arginine by the splanchnic circulation, higher doses of arginine may be required to fully restore circulating arginine levels [149]. Since it has a more favorable pharmacokinetic profile, the use of citrulline should also be considered as it is more efficient than arginine in raising systemic arginine availability [150]. Interestingly, the supplementation with vitamin D, i.e., another dietary compound that regulates immune and vascular cell function, has also been proposed for COVID-19 patients, further highlighting the potential use of nutraceuticals in treating this infection [151,152,153].

A potential concern with arginine replenishment therapies is that arginine may be channeled via maladaptive pathways (ARG1) to worsen immune and vascular cell dysfunction in COVID-19 [154,155]. In addition, arginine may elicit pleiotropic effects that aggravate cardiovascular disease [144,145,146,147,148,149,150,151,152,153,154,155,156]. In this respect, the use of ARG inhibitors may provide a more selective approach in treating COVID-19 patients. Several highly potent ARG inhibitors have been developed and their therapeutic potential has been validated in several small clinical studies. Intrabrachial infusions of an ARG inhibitor increases local forearm endothelium-dependent vasodilation in patients with familial hypercholesterolemia, type 2 diabetes, and coronary artery disease, as well as in healthy elderly subjects [157,158,159]. Similarly, the administration of a combination of ARG inhibitors in dorsal forearm skin by intradermal microdialysis significantly augments local cutaneous vasodilation in patients with arterial hypertension [160]. Importantly, ARG inhibitors are well tolerated and exhibit no reported toxicities with few non-specific actions [161,162]. Currently, two promising ARG inhibitors are used in clinical trials: Numidargistat for cancer immunotherapy and CB-280 for cystic fibrosis treatment [163]. Curiously, many comorbidities which increase the risk of infection and poor outcomes in COVID-19, such as diabetes, hypertension, cardiovascular disease, chronic kidney disease, and old age, are associated with endothelial dysfunction and high ARG activity [9,10,11,12,13,14,15,16,17,18,164]. The elevation in ARG expression in these highly vulnerable patient groups, who are likely to respond favorably to strategies targeting ARG, may explain the adverse outcomes in these patients.

The direct administration of arginine metabolites provides another therapeutic modality for treating patients with COVID-19. Inhalation of NO is under study in numerous COVID-19-related clinical trials (see [131]). These interventional studies with NO aim to reverse virus burden, bronchoconstriction, inflammation, and respiratory failure; treat and prevent progression in patients with mild and moderate disease; and act as a protective option for healthcare providers. One small study found that inhaled NO is well tolerated and might benefit pregnant patients with hypoxic respiratory failure [165], while other minor trials suggested that inhaled NO therapy may prevent the progression of hypoxic respiratory failure in spontaneously breathing COVID-19 patients [166,167]. A case report also determined that inhaled NO ameliorates dyspnea and fatigue in a single patient with idiopathic pulmonary hypertension that had been diagnosed with COVID-19 [168]. Single-center prospective studies also reported that inhaled NO increases ventilation/perfusion match in patients with severe pneumonia [169,170]. However, other studies found that inhaled NO fails to restore arterial oxygenation in COVID-19 patients with severe hypoxemia [171,172]. Similarly, a larger multicenter study showed that inhaled NO via a high-flow nasal cannula did not reduce oxygen requirements in COVID-19 patients with respiratory failure or the need for mechanical ventilation [173]. The future release of ongoing clinical trials may further clarify the utility and dosing requirements of inhaled NO in COVID-19 patients. 

The use of donor molecules provides another avenue for the delivery of NO. Numerous NO-releasing molecules that possess unique biophysical properties, half-life, and release kinetics that are dictated by specific stimuli (such as light, heat, and pH) have been developed [174]. In addition, the incorporation of NO into polymers through micelles, dendrimers, star-shaped polymers, and polymeric nanoparticles permits the liberation of NO in a more continuous fashion [175]. Recent clinical studies have also highlighted the utility of oral nitrate therapy in raising circulating levels of NO [176]. Dietary or intravenously administered inorganic nitrate is reduced to NO via the entero-salivary circulation. Nitrate supplementation with beetroot juice improves endothelial function and blood pressure in patients with hypertension and shows promise in conditions of myocardial infarction, heart failure, stroke, and pulmonary hypertension [177,178]. Epidemiological studies have also implicated low levels of the arginine metabolite homoarginine as a risk factor for cardiovascular disease [178]. In addition, several experimental studies suggest that homoarginine plays a direct protective role in the circulation, possibly by promoting NO synthesis by serving as a NOS substrate and/or an ARG inhibitor. An early phase one clinical trial in healthy volunteers found that oral supplementation with homoarginine elevates plasma homoarginine concentration without any adverse effects, paving the way for larger prospective studies in patients with cardiovascular disease [179]. Given that homoarginine levels are depressed in COVID-19 patients, the oral administration of homoarginine may be beneficial in this patient population [180].

Aside from elevating circulating levels of NO, one can also augment the biological activity of the gas. Since many of the beneficial effects of NO in the circulation are mediated by the activation of soluble guanylate cyclase (sGC) and the subsequent rise in intracellular cyclic guanosine monophosphate (cGMP), schemes targeting this NO signaling pathway may be beneficial [181]. Highly potent activators of sGC which activate the enzyme in oxidized or heme-free form have been developed. In addition, sGC stimulators that bind to the heme-containing form of sGC and potentiate the effects of endogenous NO are available. In this respect, the sGC stimulator riociguat is used to clinically treat pulmonary arterial hypertension. Finally, several clinically prescribed inhibitors of phosphodiesterase type 5, which specifically hydrolyses cGMP, are commonly used in the treatment of erectile dysfunction and pulmonary arterial hypertension and may be useful in treating the respiratory and vascular complications associated with COVID-19.

## 8. Conclusions

Emerging clinical studies have identified abnormalities in the metabolism of arginine in COVID-19 patients that results in a lower circulating level of this amino acid. Moreover, there is an increase in ARG1 expression with COVID-19 that shifts the metabolism of arginine away from the synthesis of NO towards the formation of ornithine, polyamines, and proline. This maladaptive response in arginine metabolism may contribute to COVID-19-mediated impairments in immune and vascular function. Strategies that target the loss of plasma arginine and the reprogramming of arginine metabolism and increase the bioavailability and potency of NO in COVID-19 provide promising approaches in mitigating the devastating consequences of SARS-CoV-2 infection. 

## References

[1] World Health Organization (2022). WHO Coronavirus Disease (COVID) Dashboard. http://covid19.who.int.

[2] Mukra R., Krishan K., Kanchan T. (2020). Possible modes of transmission of novel coronavirus SARS-COVID-2: A review. Acta Biomed..

[3] Berlin D.A., Gulick R.M., Martinez F.J. (2020). Severe COVID-19. N. Engl. J. Med..

[4] Gautret P., Million M., Jarrot P.A., Camion-Jau L., Colson P., Fenollar F., Leone M., la Scola B., Devaux C., Gaubert J.Y. (2020). Natural history of COVID-19 and therapeutic options. Expert Rev. Clin. Immunol..

[5] Casadevall A., Pirofski L.A. (2018). What is a host? Attributes of individual susceptibility. Infect Immun..

[6] McFadyen J.D., Stevens H., Peter K. (2020). The emerging threat of (micro) thrombosis in COVID-19 and its therapeutic implications. Circ. Res..

[7] Rodriguez P.C., Ochoa A.C., Al-Khami A.A. (2017). Arginine metabolism in myeloid cells shapes innate and adaptive immunity. Front. Immunol..

[8] Halaby M.J., McGaha T.L. (2021). Amino acid transport and metabolism in myeloid function. Front. Immunol..

[9] Yang Z., Ming X.-F. (2013). Arginase: The emerging therapeutic target for vascular oxidative stress and inflammation. Front. Immunol..

[10] Munder M. (2009). Arginase: An emerging key player in the mammalian immune system. Br. J. Pharm..

[11] Marti I Lindez A.-A., Reith W. (2021). Arginine-dependent immune responses. Cell Mol. Life Sci..

[12] Wu G., Morris S.M. (1988). Arginine metabolism: Nitric oxide and beyond. Biochem. J..

[13] Durante W. (2001). Regulation of L-arginine transport and metabolism in vascular smooth muscle cells. Cell Biochem. Biophys..

[14] Durante W., Johnson F.K., Johnson R.A. (2007). Arginase: A critical regulator of nitric oxide synthesis and vascular function. Clin. Exp. Pharmacol. Physiol..

[15] Durante W. (2013). Role of arginase in vessel wall remodeling. Front. Immunol..

[16] Yang Z., Ming X.F. (2006). Endothelial arginase: A new target in atherosclerosis. Curr. Hypertens. Rep..

[17] Pernow J., Jung C. (2013). Arginase as a potential target in the treatment of cardiovascular disease: Reversal of arginine steal?. Cardiovasc. Res..

[18] Caldwell R.W., Rodriguez P.C., Toque H.A., Narayanan S.P., Caldwell R.B. (2018). Arginase: A multifaceted enzyme important in health and disease. Physiol. Rev..

[19] Falck-Jones S., Vangeti S., Yu M., Falck-Jones R., Cagigi A., Badolati I., Österberg B., Lautenbach M.J., Åhlberg E., Lin A. (2021). Functional monocytic myeloid-derived suppressor cells increase blood but not airways and predict COVID-19 severity. J. Clin. Investig..

[20] Husson A., Brasse-Lagnel C., Fairand A., Renouf S., Lavoinne A. (2003). Argininosuccinate synthetase for the urea cycle to the citrulline-NO cycle. Eur. J. Biochem..

[21] Fostermann U., Sessa W.C. (2012). Nitric oxide synthases: Regulation and function. Eur. Heart J..

[22] Jenkinson C.P., Grody W.W., Cederbaum S.D. (1996). Comparative properties of arginase. Comp. Biochem. Physiol. B Biochem. Mol. Biol..

[23] Tabor C.W., Tabor H. (1984). Polyamines. Ann. Rev. Biochem..

[24] Durante W., Liao L., Peyton K.J., Schafer A.I. (1998). Thrombin stimulates vascular smooth muscle cell polyamine synthesis by inducing cationic amino acid transporter and ornithine decarboxylase activity. Circ. Res..

[25] Durante W., Liao L., Reyna S.V., Peyton K.J., Schafer A.I. (2000). Physiologic cyclic stretch directs L-arginine transport and metabolism to collagen synthesis in vascular smooth muscle cells. FASEB J..

[26] Durante W., Liao L., Reyna S.V., Peyton K.J., Schafer A.I. (2001). Transforming growth factor-β1 stimulates L-arginine transport and metabolism in vascular smooth muscle cells: Role in polyamine and collagen synthesis. Circulation.

[27] Kim J.H., Bugaj L.J., Oh Y.J., Bivalacqua T.J., Ryhoo S., Soucy K.G., Santhanam L., Webb A., Camara A., Sikka G. (2009). Arginase inhibition restores NOS coupling and reverses endothelial dysfunction and vascular stiffness in old rats. J. Appl. Physiol..

[28] Mossner J., Hammermann R., Racke K. (2001). Concomitant down-regulation of L-arginine transport and nitric oxide (NO) synthesis in rat alveolar macrophages by the polyamine spermine. Pulm. Pharmacol. Ther..

[29] Lee J., Rhu H., Ferrante R.J., Morris S.M., Ratan R.R. (2003). Translational control of inducible nitric oxide synthase expression by arginine can explain the arginine paradox. Proc. Natl. Acad. Sci. USA.

[30] Daghigh F., Fukuto J.M., Ash D.E. (1994). Inhibition of rat liver arginase by an intermediate in NO biosynthesis, NG-hydroxy-L-arginine: Implications for the regulation of nitric oxide biosynthesis by arginase. Biochem. Biophys. Res. Commun.

[31] Santhanam L., Lim H.K., Miriel V., Brown T., Patel M., Balanson S., Ryoo S., Anderson M., Irani K., Khanday F. (2007). Inducible NO synthase-dependent S-nitrosylation and activation of arginase1 contribute to age-related endothelial dysfunction. Circ. Res..

[32] Karupiah G., Harris N. (1995). Inhibition of viral replication by nitric oxide and its reversal by ferrous sulfate and tricarboxylic acid cycle intermediates. J. Exp. Med..

[33] Kawanishi M. (1995). Nitric oxide inhibits Epstein-Barr virus DNA replication and activation of latent EBV. Intervirology.

[34] Harris N., Buller R.M., Karupiah G. (1995). Gamma interferon-induced nitric oxide-mediated inhibition of vaccinia virus replication. J. Virol..

[35] Lowenstein C.J., Hill S.L., Lafond-Walker A., Wu J., Allen G., Landavere M., Rose N.R., Herskowitz A. (1996). Nitric oxide inhibits viral replication in murine myocarditis. J. Clin. Investig..

[36] Saura M., Zaragoza C., McMillan A., Quick R.A., Hohenadl C., Lowenstein J.M., Lowenstein C.J. (1999). An antiviral mechanism of nitric oxide: Inhibition of a viral protease. Immunity.

[37] Akerstrom S., Mousavi-Jazi M., Klingstrom J., Leijon M., Lundkvist A., Mirazimi A. (2005). Nitric oxide inhibits the replication cycle of severe acute respiratory syndrome coronavirus. J. Virol..

[38] Akerstrom S., Gunalan V., Keng C.T., Tan Y.-J., Mirazimi A. (2009). Dual effect of nitric oxide on SARS-CoV replication: Viral RNA production and palmitoylation of the S protein are affected. Virology.

[39] Shang J., Wan Y., Luo C., Ye G., Geng Q., Auerbach A., Li F. (2020). Cell entry mechanisms of SARS-CoV-2. Proc. Natl. Acad. Sci. USA.

[40] Zhou P., Yang X.L., Wang X.G., Hu B., Zhang L., Zhang W., Si H.-R., Zhu Y., Li B., Huang C.-L. (2020). A pneumonia outbreak associated with a new coronavirus of probable bat origin. Nature.

[41] Louis C.A., Mody V., Henry W.L., Reichner J.S., Albina J.E. (1999). Regulation of arginase isoforms I and II by IL-4 in cultured murine peritoneal macrophages. Am. J. Physiol..

[42] Albina J.E., Mills C.D., Barbul A., Thirkill C.E., Henry W.L., Mastrofrancesco B., Caldwell M.D. (1988). Arginine metabolism in wounds. Am. J. Physiol..

[43] Dunand-Sauthier I., Irla M., Carnesechi S., Seguin-Estevez Q., Vejnar C.E., Zdobnov E.M., Santiago-Raber M.-L., Reith W. (2014). Repression of arginase-2 expression in dendritic cells by microRNA-155 is critical for promoting T cell proliferation. J. Immunol..

[44] Munder M., Schneider H., Luckner C., Giese T., Langhans C.D., Fuentes J.M., Kropf P., Mueller I., Kolb A., Modolell M. (2006). Suppression of T cell functions by granulocyte arginase. Blood.

[45] Rotondo R., Bertolotto M., Barisione G., Astigiano S., Mandruzzato S., Ottonello L., Dallegri F., Bronte V., Ferrini S., Barbieri O. (2011). Exocytosis of azurophil and arginase-1-containing granules by activated polymorphonuclear neutrophils is required to inhibit T lymphocyte proliferation. J. Leukoc. Biol..

[46] Rodriguez P.C., Quiceno D.G., Zabaleta J., Ortiz B., Zea A.H., Piazuelo M.B., Delgado A., Correa P., Brayer J., Sotomayor E.M. (2004). Arginase I production in the tumor microenvironment by mature myeloid cells inhibits T-cell receptor expression and antigen-specific T-cell receptor responses. Cancer Res..

[47] Zea A.H., Rodriguez P.C., Culotta K.S., Hernandez C.P., DeSalvo J., Ochoa J.B., Park H., Zabaleta J., Ochoa A.C. (2004). L-Arginine modulates CD3zeta expression and T cell function in activated T lymphocytes. Cell. Immunol..

[48] Geiger R., Rieckmann J.C., Wolf T., Basso C., Feng Y., Fuhrer T., Kogadeeva M., Picotti P., Meissner F., Mann M. (2016). L-Arginine modulates T cell metabolism and enhances survival and anti-tumor activity. Cell.

[49] Fletcher M., Ramirez M.E., Sierra R.A., Raber P., Thevenot P., Al-Khami A.A., Sanchez-Pino D., Hernandez C., Wyczechowska D.D., Ochoa A.C. (2015). l-Arginine-depletion blunts antitumor T-cell responses by inducing myeloid-derived suppressor cells. Cancer Res..

[50] Taheri F., Ochao J.B., Faghiri Z., Culotta K., Park H.J., Lan M.S., Zea A.H., Ochoa A.C. (2001). L-Arginine regulates the expression of the T-cell receptor zeta chain (CD3zeta) in Jurkat cells. Clin. Cancer Res..

[51] Rodriguez P.C., Hernandez C.P., Morrow K., Sierra R., Zabaleta J., Wyczechowska D.D., Ochoa A.C. (2010). L-Arginine deprivation regulates cyclin D3 mRNA stability in human T cells by controlling HuR expression. J. Immunol..

[52] Rodriguez P.C., Quiceno D.G., Ochoa A.C. (2007). L-Arginine availability regulates T-lymphocyte cell cycle progression. Blood.

[53] Van de Velde L.A., Murray P.J. (2016). Proliferating helper T cells require Rictor/mTORC2 complex to integrate signals from limiting environmental amino acids. J. Biol. Chem..

[54] Mazzoni A., Bronte V., Visintin A., Spitzer J.H., Apolloni E., Serafini P., Zanovello P., Segal D.M. (2002). Myeloid suppressor lines inhibit T cell responses by an NO-dependent mechanism. J. Immunol..

[55] Saio M., Radoja S., Marino M., Frey A.B. (2001). Tumor-infiltrating macrophages induce apoptosis in activated CD8(+) T cells by a mechanism requiring cell contact and mediated by both the cell-associated form of TNF and nitric oxide. J. Immunol..

[56] Peranzoni E., Marigo I., Dolcetti L., Ugel S., Sonda N., Taschin E., Mantelli B., Bronte V., Zanovello P. (2007). Role of arginine metabolism in immunity and immunopathology. Immunobiol.

[57] Vig M., Srivastava S., Kandal U., Sade H., Lewis V., Sarin A., George A., Bal V., Durdik J.M., Rath S. (2004). Inducible nitric oxide synthase in T cells regulates T cell death and immune memory. J. Clin. Investig..

[58] De Jonge W.J., Kwikkers K.L., te Velde A.A., van Deventer S.J.H., Nolte M.A., Mebius R.E., Ruijter J.M., Lamers M.C., Lamers W.H. (2002). Arginine deficiency affects early B cell maturation and lymphoid organ development in transgenic mice. J. Clin. Investig..

[59] Saini A.S., Shenoy G.N., Rath S., Bal V., George A. (2014). Inducible nitric oxide synthase is a major intermediate in signaling pathways for the survival of plasma cells. Nat. Immunol..

[60] Park K.G., Hayes P.D., Garlick P.J., Sewell H., Eremin O. (1991). Stimulation of lymphocyte natural cytotoxicity by L-arginine. Lancet.

[61] Brittenden J., Park K.G., Heys S.D., Ross C., Ashby J., Ah-See A.K., Eremin O. (1994). L-arginine stimulates host defenses in patients with breast cancer. Surgery.

[62] Reynolds J.V., Daly J.M., Zhang S., Evantash E., Shou J., Sigal R., Ziegler M.M. (1988). Immunomodulatory mechanisms of arginine. Surgery.

[63] Lamas B., Vergnaud-Gauduchon J., Goncalves-Mendes N., Perche O., Rossary A., Vasson M.-P., Farges M.-C. (2012). Altered functions of natural killer cells in response to L-arginine availability. Cell. Immunol..

[64] Faruke K., Burd P.R., Horvath-Arcidicono J.A., Hori K., Mostowski H., Bloom E.T. (1999). Human NK cells express endothelial nitric oxide synthase, and nitric oxide protects them from activation-induced cells death by regulating expression of TNF-alpha. J. Immunol..

[65] Jyothi M.D., Khar A. (2000). Interleukin-2-induced nitric oxide synthase and nuclear factor-kappaB activity in activated natural killer cells and the production of interferon-gamma. Scand. J. Immunol..

[66] Oberlies J., Watzl C., Giese A.T., Luckner C., Kropf P., Muller I., Ho A.D., Munder M. (2009). Regulation of NK cell function by human granulocyte arginase. J. Immunol..

[67] Goh C.C., Roggerson K.M., Lee H.-C., Golden-Mason L., Rosen H.R., Hahn Y.S. (2016). Hepatitits C virus-induced myeloid-derived suppressor cells suppress NK cell IFN-γ production by altering cellular metabolism via arginase-1. J. Immunol..

[68] Xu Y., Arora R.C., Hiebert B.M., Lerner B., Szwajcer A., McDonald K., Rigatto C., Komenda P., Sood M., Tangri N. (2014). Non-invasive endothelial function testing and the risk of adverse outcomes: A systematic review and meta-analysis. Eur. Heart J. Cardiovasc. Imaging.

[69] Lundberg J.O., Gladwin M.T., Weitzberg E. (2015). Strategies to increase nitric oxide signalling in cardiovascular disease. Nat. Rev. Drug. Discov..

[70] Elms S., Chen F., Wang Y., Qian J., Askari B., Yu Y., Pandey D., Iddings J., Caldwell R.B., Fulton D.J.R. (2013). Insights into the arginine paradox: Evidence against the importance of subcellular localization of arginase and eNOS. Am. J. Physiol. Heart Circ. Physiol..

[71] Mahdi A., Kovames O., Pernow J. (2020). Improvement in endothelial function in cardiovascular disease—is arginase the target?. Int. J. Cardiol..

[72] Zhu W., Chandrsekharan U.M., Bandyopadhyay S., Morris S.M., DiCorleto P.E., Kashyap V.S. (2010). Thrombin induces endothelial arginase through AP-1 activation. Am. J. Physiol. Cell Physiol..

[73] Lewis C., Zhu W., Pavkov M.L., Kinney C.M., DiCorleto P.E., Kashyap V.S. (2008). Arginase blockade lessens endothelial dysfunction after thrombosis. Vasc. Surg..

[74] Ryoo S., Gupta G., Benjo A., Lim H.K., Camara A., Sikkha G., Lim H.K., Sohi J., Santhanam L., Soucy K. (2008). Endothelial arginase II: A novel target for the treatment of atherosclerosis. Circ. Res..

[75] Johnson F.K., Peyton K.J., Liu X.M., Azam M.A., Shebib A.R., Johnson R.A., William D. (2015). Arginase promotes endothelial dysfunction and hypertension in obese rats. Obesity.

[76] Johnson F.K., Johnson R.A., Peyton K.J., Shebib A.R., Durante W. (2013). Arginase promotes skeletal muscle arteriolar endothelial dysfunction in diabetic rats. Front. Immunol..

[77] Romero M.J., Platt D.H., Yawfik H.E., Labazi M., El-Remessy A.B., Bartoli M., Caldwell R.B., Caldwell R.W. (2008). Diabetes-induced coronary vascular dysfunction involves increased arginase activity. Circ. Res..

[78] Johnson F.K., Johnson R.A., Peyton K.J., Durante W. (2005). Arginase inhibition restores arterial endothelial dysfunction in Dahl rats with salt-induced hypertension. Am. J. Physiol. Regul. Integr. Comp. Physiol..

[79] Johnson F.K., Durante W., Craig T., Peyton K.J., Myers J.G., Stewart R.M. (2010). Vascular arginase contributes to arteriolar endothelial dysfunction in a rat model of hemorrhagic shock. J. Trauma..

[80] Jung C., Gonon A.T., Sjoquist P.O., Lundberg J.O., Pernow J. (2010). Arginase inhibition mediates cardioprotection during ischemia-reperfusion. Cardiovasc. Res..

[81] Steppan J., Tran H.T., Bead V.R., Oh Y.J., Sikka G., Bivalacqua T.J., Burnett A.L., Berkowitz D.E., Santhanam L. (2016). Arginase inhibition reverses endothelial dysfunction, pulmonary hypertension, and vascular stiffness in transgenic sickle cell mice. Anesth. Analg..

[82] Demougeot C., Prigent-Tessier A., Marie C., Berthelot A. (2005). Arginase inhibition reduced endothelial dysfunction and blood pressure rising in spontaneously hypertensive rats. J. Hypertens..

[83] Cho W., Lee C., Kang M., Huang Y., Giordano F.J., Lee P.J., Trow T.K., Homer R.J., Sessa W.C., Elias J.A. (2013). IL-13 receptor α_2_-arginase 2 pathway mediates IL-13-iinduced pulmonary hypertension. Am. J. Physiol. Lung Cell. Mol. Physiol..

[84] Wei L.H., Wu G., Morris S.M., Ignarro L.J. (2001). Elevated arginase 1 expression in rat aortic smooth muscle cells increases cell proliferation. Proc. Natl. Acad. Sci. USA.

[85] Peyton K.J., Ensenat D., Azam M.A., Keswani A.N., Kanna S., Liu X.-M., Wang H., Tulis D.A., Durante W. (2009). Arginase promotes neointima formation in rat injured carotid arteries. Arter. Thromb. Vasc. Biol..

[86] Bagnost T., Ma L., da Silva R.F., Rezakhaniha R., Houdayer C., Stergiopulos N., André C., Guillaume Y., Berthelot A., Demougeot C. (2010). Cardiovascular effects of arginase inhibition in spontaneously hypertensive rats with fully developed hypertension. Cardiovasc. Res..

[87] Bhatta Y., Yao L., Haque H.A., Shatanawi A., Xu Z., Caldwell R.B., Caldwell R.W. (2015). Angiotensin II-induced arterial thickening, fibrosis and stiffening involve elevated arginase function. PLoS ONE.

[88] Grasemann H., Dhaliwal R., Ivanovska J., Kantores C., McNamara P.J., Scott J.A., Belik J., Jankov R.P. (2015). Arginase inhibition prevents bleomycin-induced pulmonary vascular remodeling, and collagen deposition in neonatal rat lungs. Am. J. Physiol. Lung Cell. Mol. Physiol..

[89] Cowburn A.S., Crosby A., Macias D., Branco C., Colaco R.D., Southwood M., Toshner M., Alexander L.E.C., Morrell N., Chilvers E. (2016). HIF2α-arginase axis is essential for the development of pulmonary hypertension. Proc. Natl. Acad. Sci. USA.

[90] Bhatta A., Yao L., Xu Z., Toque H.A., Chen J., Atawia R.T., Fouda A.Y., Bagi Z., Lucas R., Caldwell R.B. (2017). Obesity-induced vascular dysfunction and arterial stiffening requires endothelial arginase 1. Cardiovasc. Res..

[91] Cui S., Chen S., Li X., Liu S., Wang F. (2020). Prevalence of venous thromboembolism in patients with severe novel coronavirus pneumonia. J. Thromb. Haemost..

[92] Klok F.A., Kruip M.J.H.A., van der Meer N.J.M., Arbous M.S., Gommers D.A.M.P.J., Kant K.M., Kaptein F.H.J., van Paassen J., Stals M.A.M., Huisman M.V. (2020). Confirmation of the high cumulative incidence of thrombotic complications in critically ill ICU patients with COVID-19: An updated analysis. Thromb. Res..

[93] Wichmann D., Sperhake J.P., Lutgehetmann M., Steurer S., Edler C., Heinemann A., Heinrich F., Mushumba H., Kniep I., Schröder A.S. (2020). Autopsy findings and venous thromboembolism in patients with COVID-19: A prospective cohort study. Ann. Intern. Med..

[94] Zhou F., Yu T., Du R., Fan G., Liu Y., Liu Z., Xiang J., Wang Y., Song B., Gu X. (2020). Clinical course and risk factors for mortality of adult inpatients with COVID-19 in Wuhan, China: A retrospective cohort study. Lancet.

[95] Yang X., Yang Q., Wang Y., Wu Y., Xu J., Yu Y., Shange Y. (2020). Thrombocytopenia and its association with mortality in patients with COVID-19. J. Thromb. Haemostas.

[96] Lippi G., Favoloro E.J. (2020). D-dimer is associated with severity of coronavirus disease 2019: A pooled analysis. J. Thromb. Haemostas..

[97] Lippi G., Phlebani M., Henry B.M. (2020). Thrombocytopenia is associated with severe coronavirus disease 2019 (COVID-19) infections: A meta-analysis. Clin. Chem. Acta.

[98] Modin D., Claggett B., Sindet-Pedersen C., Lassen M.C.H., Skaarup K.G., Jensen J.U.S., Fralick M., Schou M., Lamberts M., Gerds T. (2020). Acute COVID-19 and the incidence of ischemic stroke and acute myocardial infarction. Circulation.

[99] Oxley T.J., Mocco J., Majidi S., Kellner C.P., Shoirah H., Singh I.P., De Leacy R.A., Shigematsu T., Ladner T.R., Yaeger K.A. (2020). Large-vessel stroke as a presenting feature of COVID-19 in the young. N. Engl. J. Med..

[100] Gonzalez-Urquijo M., Gonzalez-Rayes J.M., Castro-Varela A., Hinojosa-Gonzalez D.E., Ramos-Cazares R.E., Vazquez-Garza E., Paredes-Vazquez J.G., Castillo-Perez M., Jerjes-Sanchez C., Fabiani M.A. (2021). Unexpected arterial thrombosis and acute limb ischemia in COVID-19 patients. Results from the Ibero-Latin American acute arterial thrombosis registry in COVID-19: (ARTICO-19). Vascular.

[101] Ackermann M., Verleden S.E., Kuehnel M., Havervich A., Welte T., Laenger F., Vanstapel A., Werlein C., Stark H., Tzankov A. (2020). Pulmonary vascular endothelialitis, thrombosis, and angiogenesis, in COVID-19. N. Engl. J. Med..

[102] Bradley B.T., Maioli H., Johnston R., Chaudhary I., Fink S.L., Xu H., Najafian B., Deutsch G., Lacy J.M., Williams T. (2020). Histopathology and ultrastructural findings of fatal COVID-19 infections in Washington State: A case series. Lancet.

[103] Gattinoni L., Chiumello D., Caironi P., Busana M., Romitti F., Brazzi L., Camporota L. (2020). COVID-19 pneumonia: Different respiratory treatments for different phenotypes?. Intensive Care Med..

[104] Rapkiewicz A.V., Mai X., Carsons S.E., Pittaluga S., Kleiner D.E., Berger J.S., Thomas S., Adler N., Charytan D., Gasmi B. (2020). Megakaryocytes and platelet-fibrin thrombi characterize multi-organ thrombosis at autopsy in COVID-19: A case series. EClinical.

[105] Viola F., Pignatelli P., Cammisotto V., Bartimoccia S., Carnevale R., Nocella C. (2021). COVID-19 and thrombosis: Clinical features, mechanism of disease, and therapeutic implications. Kardiol. Pol..

[106] Gu S.X., Tyagi T., Jain K., Gu V.W., Lee S.H., Hwa J.M., Kwan J.M., Krause D.S., Lee A.I., Halene S. (2021). Thrombocytopathy and endothelialiopathy: Crucial contributors to COVID-19 thromboinflammation. Nat. Rev. Cardiol..

[107] Nicosia R.F., Ligresti G., Caporarello N., Akilesh S., Ribatti D. (2021). COVID-19 Vasculopathy: Mounting evidence for an indirect mechanism of endothelial injury. Am. J. Pathol..

[108] Ma Z., Yang K.Y., Huang Y., Liu K.O. (2022). Endothelial contribution to COVID-19: An update on mechanisms and therapeutic implications. J. Mol. Cell. Cardiol..

[109] Prasad M., Leion M., Lerman L.O., Lerman A. (2021). Viral endothelial dysfunction: A unifying mechanism for COVID-19. Mayo. Clin. Proc..

[110] Varga Z., Flammer A.J., Steiger P., Haberecker M., Andermatt R., Zinkernagel A.S., Mehra M.R., Schuepbach R.A., Ruschitzka F., Moch H. (2020). Endothelial cell infection and endothelialitis in COVID-19. Lancet.

[111] Fox S.E., Lameira F.S., Rinker E.B., Vander Heide R.S. (2020). Cardiac endothelialitis and multisystem inflammatory syndrome after COVID-19. Ann. Intern. Med..

[112] Carnevale S., Beretta P., Morbini P. (2021). Direct endothelial damage and vasculitis due to SARS-CoV-2 in small bowel submucosa of CIVD-19 patients with diarrhea. J. Med. Virol..

[113] Negrini S., Guadagno A., Greco M., Parodi A., Burlando M. (2020). An unusual case of bullous haemorrhagic vasculitis in a COVID-19 patient. J. Eur. Acad. Dermatol. Venereol..

[114] Escher R., Breakey N., Lammle B. (2020). Severe COVID-19 infection associated with endothelial activation. Thromb. Res..

[115] Fraser D.D., Patterson E.K., Daley M., Cepinskas G., on behalf of the Lawson COVID-19 Study Team (2021). Case report: Inflammation and endothelial injury profiling of COVID-19 pediatric multisystem inflammatory syndrome (MIS-C). Fronti. Pediatr..

[116] Crippa S., Kagi G., Graf L., Meyer Sauteur P.M., Kohler P. (2020). Stroke in young adult with mild COVID-19 suggesting endothelialitis. New Microbes New Infect..

[117] Guervilly C., Burtey S., Sabatier F., Cauchois R., Lano G., Abdili E., Daviet F., Arnaud L., Brunet P., Hraiech S. (2020). Circulating endothelial cells as a marker of endothelial injury in severe COVID-19. J. Infect. Dis..

[118] Neri T., Nieri D., Celi A. (2020). P-selectin blockade in COVID-19-related ARDS. Am. J. Physiol. Lung Cell. Mol. Physiol..

[119] Smadja D.M., Guerin C.L., Chocron R., Yatim N., Boussier J., Gendron N., Khider L., Hadjadj J., Goudot G., Debuc B. (2020). Angiopoietin-2 as a marker of endothelial activation is a good predictor factor for intensive care unit admission of COVID-19 patients. Angiogenesis.

[120] Spadaro S., Fogagnolo A., Campo G., Zucchetti O., Verri M., Ottaviani I., Tunstall T., Grasso S., Scaramuzzo V., Murgolo F. (2021). Markers of endothelial and epithelial pulmonary injury in mechanically-ventilated COVID-19 ICU patients. Crit. Care.

[121] Sabioni L.R., Tibirica E., Lamas C.C., Amorim G.D., De Lorenzo A. (2020). Systemic microvascular dysfunction in COVID-19. Am. J. Cardiovasc. Dis..

[122] Sabioni L., De Lorenzo A., Lamas C., Muccillo F., Castro-Faria-Neto H.C., Estato V., Tibirica E. (2021). Systemic microvascular endothelial dysfunction and disease severity in COVID-19 patients: Evaluation by laser doppler perfusion monitoring and cytokine/chemokine analysis. Microvasc. Res..

[123] Ratchford S.M., Stickford J.L., Province V.M., Stute N., Augenreich M.A., Koontz L.K., Bobo L.K., Stickford A.S.L. (2021). Vascular alterations among young adults with SARS-CoV-2. Am. J. Physiol. Heart Circ. Physiol..

[124] Zhang X., Sun D., Song J.W., Zullo J., Lipphardt M., Coneh-Gould L., Goligorsky M.S. (2019). Endothelial cell dysfunction and the glycocalyx—a vicious circle. Matrix Biol..

[125] Fraser D.D., Patterson E.K., Slessarev M., Gill S.E., Martinc C., Daley M., Miller M.R., Patel M.A., Santos C.C.D., Bosma K.J. (2020). Endothelial injury and glycocalyx degradation in critically ill coronavirus disease 2019 patients: Implications for microvascular platelet aggregation. Crit. Care Explor..

[126] Du Preez H.N., Aldous C., Hayden M.R., Kruger H.G., Lin J. (2022). Pathogenesis of COVID-19 described through the lens of undersulfated and degraded epithelial and endothelial glycocalyx. FASEB J..

[127] Chang R., Mamun A., Dominic A., Le N.T. (2020). SARS-CoV-2 mediated endothelial dysfunction. The potential role of chronic oxidative stress. Front. Physiol..

[128] Adebayo A., Varzideh F., Wilson S., Gambardella J., Eacobacci M., Jankauskas S.S., Donkor K., Kansakar U., Trimarco V., Mone P. (2021). L-Arginine and COVID-19: An update. Nutrients.

[129] Guimaraes L.M.F., Rossini C.V.T., Lameu C. (2021). Implications of SARS-CoV-2 infection on eNOS and iNOS activity: Consequences for the respiratory and vascular systems. Nitric. Oxide..

[130] Fang W., Jiang J., Su L., Shu T., Liu H., Lai S., Ghiladi R.A., Wang J. (2021). The role of NO in COVID-19 and potential therapeutic strategies. Free Radic. Biol. Med..

[131] Alamdari D.H. (2020). Application of methylene blue-vitamin C-N-acetyl cysteine for treatment of critical ill COVID-19 patients, report of a phase -I clinical trial. Eur. J. Pharmacol..

[132] Derakhshani A., Hemmat N., Asadzadeh Z., Ghaseminia M., Shadbad M.A., Jadideslam G., Silvestris N., Racanelli V., Baradaran B. (2021). Arginase 1 (Arg1) as an up-regulated gene in COVID-19 patients: A promising marker of COVID-19 immunopathy. J. Clin. Med..

[133] Hemmat N., Derakhshani A., Baghi H.B., Silvestris N., Baradaran B., De Summa S. (2020). Neutrophils, crucial, or harmful immune cells involved in coronavirus infection: A bioinformatics study. Front. Genet..

[134] Syrimi E., Fennell E., Richter A., Vrljicak P., Stark R., Ott S., Murray P.G., Al-Abadi E., Chikermane A., Dawson P. (2021). The immune landscape of SARS-CoV-2-associated multisystem inflammatory syndrome in children (MIS-C) from acute disease to recovery. iScience.

[135] Reizine F., Lesouhaitier M., Gregoire M., Pinceaux K., Gacouin A., Maamare A., Painvin B., Camus C., le Tulzo Y., Tattevin P. (2021). SARS-CoV-2-induced ARDS associates with MDSC expansion, lymphocyte dysfunction, and arginine shortage. J. Clin. Immunol..

[136] Dean M.J., Ochoa J.B., Sanchez-Pino M.D., Zabaleta J., Garai J., Del Valle L., Wyczechowska D., Baiamonte L.B., Philbrook P., Majumder R. (2021). Severe COVID-19 is characterized by an impaired type I interferon response and elevated levels of arginase producing granulocytic myeloid derived suppressor cells. Front. Immunol..

[137] Sacchi A., Grassi G., Notari S., Gili S., Bordoni V., Tartaglia E., Casetti R., Cimini E., Mariotti D., Garotto G. (2021). Expansion of myeloid derived suppressor cells contributes to platelet activation by L-arginine deprivation during SARS-CoV-2 infection. Cells.

[138] D’Alessandro A., Thomas T., Akpan I.J., Reisz J.A., Cendali F.I., Gamboni F., Nemkov T., Thangaraju K., Katneni U., Tanaka K. (2021). Biological and clinical factors contributing to the metabolic heterogeneity of hospitalized patients with and without COVID-19. Cells.

[139] She B., Yi X., Sun Y., Bi X., Du J., Zhang C., Quan S., Zhang F., Sun R., Qian L. (2020). Proteomic and metabolomic characterization of COVID-19 patient sera. Cell.

[140] Wu P., Chen D., Ding W., Wu P., Hou H., Bai Y., Zhou Y., Li K., Xiang S., Liu P. (2021). The trans-omics landscape of COVID-19. Nat. Commun..

[141] Rees C.A., Rostade C.A., Mantus G., Anderson E.J., Chahroudi A., Jaggi P., Wrammert J., Ochoa J.B., Ochoa A., Basu R.K. (2021). Altered amino acid profile in patients with SARS-CoV-2 infection. Proc. Natl. Acad. Sci. USA.

[142] Tang W.H.W., Wang Z., Cho L., Brennan D.M., Hazen S.L. (2009). Diminished global arginine bioavailability and increased arginine catabolism as metabolic profile of increased cardiovascular risk. J. Am. Coll. Cardiol..

[143] Canzano P., Brambilla M., Porro B., Cosentino N., Tortorici E., Vicini S., Poggio P., Cascella A., Pengo M.F., Veglia F. (2021). Platelet and endothelial activation as potential mechanisms behind the thrombotic complications of COVID-19 patients. J. Am. Coll. Cardiol. Basic Trans Sci..

[144] Wu G., Meininger C.J. (2000). Arginine nutrition and cardiovascular function. J. Nutr..

[145] Durante W. (2020). Amino acid in circulatory function and health. Adv. Exp. Med. Biol..

[146] Grimes J.M., Khan S., Badeaux M., Rao R.M., Rowlinson S.W., Carvajal R.D. (2021). Arginine depletion as a therapeutic approach for patients with COVID-19. Int. J. Infect. Dis..

[147] Melano I., Kuo L.-L., Lo Y.-C., Sung P.-W., Tien N., Su W.-C. (2021). Effects of basic amino acids and their derivatives on SARS-CoV-2 and influenza-A virus infection. Viruses.

[148] Fiorentino G., Coppola A., Izzo R., Annunziata A., Bernardo M., Lombardi A., Trimarco V., Santulli G., Trimarco B. (2021). Effects of adding L-arginine to standard therapy with COVID-19: A randomized, double-blind, placebo-controlled, parallel-group trial. Results of the first interim analysis. EClinicalMedicine.

[149] McNeal C.J., Meininger C.J., Wilborn C.D., Tekwe C.D., Wu G. (2018). Safety of dietary supplementation with arginine in adult humans. Amino Acids.

[150] Schwedhelm E., Maas R., Freese R., Jung D., Lukas Z., Jumbrecina A., Spickler W., Schulze F., Böger R.H. (2008). Pharmacokinetic and pharmacodynamic properties of oral L-citrulline and L-arginine: Impact on nitric oxide metabolism. Br. J. Clin. Pharmacol..

[151] Murdaca G., Pioggia G., Negrini S. (2020). Vitamin D and COVID-19: An update on evidence and potential therapeutic implications. Clin. Mol. Allergy.

[152] Murdaca G., Tonacci A., Negrini S., Greco M., Borro M., Puppo F., Gangemi S. (2019). Emerging role of vitamin D in autoimmune diseases: An update on evidence and therapeutic implications. Autoimmun. Rev..

[153] Zhang J., McCullough P.A., Tecson K.M. (2020). Vitamin D deficiency in association with endothelial dysfunction: Implications for patients with COVID-19. Rev. Cardiovasc. Med..

[154] Blum A., Hathaway L., Mincemoyer R., Schenke W.H., Kirby M., Csako G., Waclawiw M.A., Panza J.A., Cannon I.R.O. (2000). Oral L-arginine in patients with coronary artery disease on medical management. Circulation.

[155] Wilson A.M., Harada R., Nair N., Balasubramanian N., Cooke J.P. (2003). L-arginine supplementation in peripheral artery disease: No benefit and possible harm. Circulation.

[156] Scalera F., Closs E.I., Flick E., Martens-Lobenhoffer J., Boissel J.P., Lendeckel U., Heimburg A., Bode-Böger S.M. (2009). Paradoxical effect of L-arginine: Acceleration of endothelial cell senescence. Biochem. Biophys. Res. Commun..

[157] Kovamees O., Shemyakin A., Eriksson M., Angelin B., Pernow J. (2016). Arginase inhibition improves endothelial function with familial hypercholesterolemia irrespective of their cholesterol level. J. Intern. Med..

[158] Kovamees O., Shemyakin A., Checa A., Wheelock C.E., Lundberg J.O., Ostenson C.-G., Pernow J. (2016). Arginase inhibition improves microvascular endothelial function in patients with type 2 diabetes mellitus. J. Clin. Endocrinol. Metab..

[159] Madhi A., Pernow J., Kovamees O. (2019). Arginase inhibition improves endothelial function in age-dependent manner in healthy elderly humans. Rejuvenation Res..

[160] Holowatz L.A., Kenney W.L. (2007). Up-regulation of arginase activity contributes to attenuated reflex cutaneous vasodilation in hypertensive humans. J. Physiol..

[161] Reid K.M., Tsung A., Kaizu T., Jeyabalan G., Ikeda A., Shao L., Wu G., Murase N., Geller D.A. (2007). Liver I/R injury is improved by the arginase inhibitor N(omega)-hydroxy-nor-L-arginine (nor-NOHA). Am. J. Physiol. Gastrointest. Liver Physiol..

[162] Huynh H.H., Harris E.E., Chin-Dusting J.F.P., Andrews L.K. (2009). The vascular effects of different arginase inhibitors in rat isolated aorta and mesenteric arteries. Br. J. Pharmacol..

[163] Borek B., Gajda T., Golebiowski A., Blaszczyk R. (2020). Boronic acid-based arginase inhibitors in cancer immunotherapy. Bioorg. Med. Chem..

[164] Ejaz H., Alsrhani A., Zafar A., Javed H., Junaid K., Abdalla A.E., Abosalif K.O., Ahmed Z., Younas S. (2020). COVID-19 and comorbidities: Deleterious impact on infected patients. J. Infect. Public Health.

[165] Safaee Fakhr B., Wiegand S.B., Pinciroli R., Gianni S., Morais C.C.A., Ikeda T., Miyazaki Y., Marutani E., Di Fenza R., Larson G. (2020). High concentrations of nitric oxide inhalation therapy in pregnant patients with severe coronavirus disease 2019 (COVID-19). Obstet. Gynecol..

[166] Weigand S.B., Safaee Fakhr B., Carroll R.W., Zapol W.M., Kacmarek R.M., Berra L. (2020). Rescue treatment with high-dose gaseous nitric oxide in spontaneously breathing patients with severe coronavirus disease 2019. Crit. Care Explor..

[167] Safaee Fakhr B., Di Fenza R., Gianni S., Wiegand S.B., Miyazaki Y., Araujo C.C., Gibson L.E., Chang M.G., Mueller A.L., Rodriguez-Lopez J.M. (2021). Inhaled high dose nitric oxide is a safe and effective respiratory treatment in spontaneous breathing hospitalized patients with COVID-19 pneumonia. Nitric Oxide.

[168] Zamanian R.T., Pollack C.V., Gentile M.A., Rahid M., Fox J.C., Mahaffe K.W., Perez V.D.J. (2020). Outpatient inhaled nitric oxide in a patient with vasoreactive idiopathic pulmonary arterial hypertension and COVID-19 infection. Am. J. Respir. Crit. Care Med..

[169] Abou-Arab O., Huette P., Debouvries F., Dupont H., Jounieaux V., Mahjoub Y. (2020). Inhaled nitric oxide for critically ill COVID-19 patients: A prospective study. Crit. Care.

[170] Ziehr D.R., Alladina J., Wolf M.E., Brait K.L., Malhotra A., La Vita C., Berra L., Hibbert K.A., Hardin C.C. (2021). Respiratory physiology of prone positioning with and without inhaled nitric oxide across the coronavirus disease 2019 acute respiratory distress syndrome severity spectrum. Crit. Care Explor..

[171] Tavazzi G., Marco P., Mongodi S., Dammassa V., Romito G., Mojoli F. (2020). Inhaled nitric oxide in patients admitted to intensive care unit with COVID-19 pneumonia. Crit. Care.

[172] Ferrari M., Santini A., Protti A., Andreis D.T., Iapichino G., Castellani G., Rendiniello V., Costantini E., Cecconi M. (2020). Inhaled nitric oxide in mechanically ventilated patients with COVID-19. J. Crit. Care.

[173] Chandel A., Patolia S., Ahmad K., Aryal S., Brown A.W., Sahjwani D., Khangoora V., Shlobin O.A., Cameron P.C., Singhal A. (2021). Inhaled nitric oxide via high-flow nasal cannula in patients with acute respiratory failure related to COVID-19. Clin. Med. Insights. Circ. Respir. Pulm. Med..

[174] Ma T., Zhang Z., Chen Y., Su H., Deng X., Liu X., Fan Y. (2021). Delivery of nitric oxide in the cardiovascular system: Implications for clinical diagnosis and therapy. Int. J. Mol. Sci..

[175] Yang C., Jeong S., Ku S., Lee K., Park M.H. (2018). Use of gasotransmitters for the controlled release of polymer-based nitric oxide carriers in medical applications. J. Control. Release.

[176] Lundberg J.O., Weitzberg E., Gladwin M.T. (2008). The nitrate-nitrite-nitric oxide pathway in physiology and therapeutics. Nat. Rev. Drug. Discov..

[177] Kapil V., Khambata R.S., Robertson A., Caulfield M.J., Ahluwalia A. (2015). Dietary nitrate provides sustained blood pressure lowering in hypertensive patients: A randomized phase 2, double-blind, placebo-controlled study. Hypertension.

[178] Karetnikova E.S., Jarzebska N., Markov A.G., Weiss N., Lentz S.R., Rodionov R.N. (2019). Is homoarginine a protective cardiovascular risk factor?. Arter. Thromb. Vasc. Biol..

[179] Atzler D., Schonhoff M., Cordts K., Ortland I., Hoppe J., Hummel F.C., Gerloff C., Jaehde U., Jagodzinski A., Böger R.H. (2016). Oral supplementation with L-homoarginine in young volunteers. Br. J. Clin. Pharmacol..

[180] Jud P., Gressenberger P., Muster V., Avian A., Meinitzer A., Strohmaier H., Sourij H., Raggam R.B., Stradner M.H., Demel U. (2021). Evaluation of endothelial dysfunction and inflammatory vasculopathy after SARS-CoV-2 infection—a cross sectional study. Front. Cardiovasc. Med..

[181] Farah C., Michel L.Y.M., Balligand J.-L. (2018). Nitric oxide signalling in cardiovascular health and disease. Nat. Rev. Cardiol..

